# Wavelet Imaging on Multiple Scales (WIMS) reveals focal adhesion distributions, dynamics and coupling between actomyosin bundle stability

**DOI:** 10.1371/journal.pone.0186058

**Published:** 2017-10-19

**Authors:** Tim Toplak, Benoit Palmieri, Alba Juanes-García, Miguel Vicente-Manzanares, Martin Grant, Paul W. Wiseman

**Affiliations:** 1 Department of Physics, McGill University, Montréal, Québec, Canada; 2 Universidad Autonoma de Madrid School of Medicine/IIS-Princesa Diego de Leon, Madrid, Spain; 3 Department of Chemistry, McGill University, Montréal, Québec, Canada; University of California Berkeley, UNITED STATES

## Abstract

We introduce and use Wavelet Imaging on Multiple Scales (WIMS) as an improvement to fluorescence correlation spectroscopy to measure physical processes and features that occur across multiple length scales. In this study, wavelet transforms of cell images are used to characterize molecular dynamics at the cellular and subcellular levels (i.e. focal adhesions). We show the usefulness of the technique by applying WIMS to an image time series of a migrating osteosarcoma cell expressing fluorescently labelled adhesion proteins, which allows us to characterize different components of the cell ranging from optical resolution scale through to focal adhesion and whole cell size scales. Using WIMS we measured focal adhesion numbers, orientation and cell boundary velocities for retraction and protrusion. We also determine the internal dynamics of individual focal adhesions undergoing assembly, disassembly or elongation. Thus confirming as previously shown, WIMS reveals that the number of adhesions and the area of the protruding region of the cell are strongly correlated, establishing a correlation between protrusion size and adhesion dynamics. We also apply this technique to characterize the behavior of adhesions, actin and myosin in Chinese hamster ovary cells expressing a mutant form of myosin IIB (1935D) that displays decreased filament stability and impairs front-back cell polarity. We find separate populations of actin and myosin at each adhesion pole for both the mutant and wild type form. However, we find these populations move rapidly inwards toward one another in the mutant case in contrast to the cells that express wild type myosin IIB where those populations remain stationary. Results obtained with these two systems demonstrate how WIMS has the potential to reveal novel correlations between chosen parameters that belong to different scales.

## Introduction

Fluorescence fluctuation techniques [1, 2] have been extended to the fluorescence imaging domain, such as image correlation spectroscopy, which analyzes the intensity fluctuations in space and time from a fluorescence image time series. Spatial correlation analysis of the images of cells can be used to measure the surface density and aggregation state distributions of fluorescently labeled receptors [3, 4]. Variants have been developed to quantify the colocalization of different species tagged with fluorophores of different colors [5], to measure transport properties of biomolecules within the cell membrane and in the cytosol over different time scales [6, 7], and to separate photophysical and transport contributions to the correlation function through a Fourier space representation [8]. Analogous developments in imaging fluorescence correlation spectroscopy use time domain correlation functions calculated from an image series to map diffusion coefficients and concentrations [9].

Spatio-temporal image correlation spectroscopy was introduced to analyze both space and time fluctuations and measure the velocity and diffusion coefficients of fluorescently labeled molecules across multiple regions of cells [10]. A space-time-dependent correlation function is calculated from the images and is fitted to theoretical models to measure local flows and diffusivities of the tagged molecules. Despite its successes, information is obtained on the molecular scale dynamics but not the macroscopic cell structural scale. Also sharp intensity changes at cell boundaries can perturb the space-time correlation functions. Thus, a method that can span multiple scales is desired.

In this paper, we introduce a new technique called wavelet imaging on multiple scales (WIMS). Wavelets have been applied in a variety of fields such as computer science and geophysics [11]. Their transforms are mathematical operations that separate different length/time scales in a data set and retain position/time information [12]. In contrast to Fourier transforms, which are powerful when used to analyze linear periodic systems, wavelet transforms are better suited when applied to study complex heterogeneous systems that are non-stationary. Cells and tissues are examples of systems that exhibit such complexity. The ability of wavelets to distinguish between different components as well as localize the time and/or space of an object/event is what differentiates it from Fourier techniques. Many types of functions qualify as wavelets, giving great flexibility in applications to identify different types of structures by using different wavelet functions. Wavelets can form either complete or overcomplete basis sets, depending on their application. The use of overcomplete bases involves an increase in numerical effort when calculating wavelet transforms. However, this increase is not a significant concern given the current state of computer processing power.

Previously, wavelets have garnered interest as a way to deconvolve images to detect hidden structures that have been in some way obstructed [13–15]. Biophysical applications of wavelets include a time series approach that examined single-molecule thermal fluctuations switching between internal states [16]. Another application was as a faster alternative to fitting the centroid of the point spread function [17]. Also, wavelet transforms have been used to characterize functional signalling domains by their size from Förster resonance energy transfer images [18]. Recently, non circularly symmetric wavelets have been devised to track the multi-orientational retinal vasculature in an image series [19].

Cells (10^4^–10^6^ nm) are made up of multiple components that vary in scale by orders of magnitude. These different organizational components include proteins (5–10 nm), protein complexes (10–20 nm) and focal adhesions (50–1000 nm). Optical resolution limits for conventional widefield optical microscopy and total internal reflection fluorescence microscopy reach a resolution of ∼250 nm, while super-resolution microscopy methods can reach a limit of between ∼20–50 nm.

There have been a variety of methods developed to investigate focal adhesions in cells [20–24]. In an effort to work around the changing morphology, Möhl et al. [20] transformed each image of an irregularly shaped cell to fit into an unvarying circle. This mapping was used to look at the fluorescent protein distribution and dynamics, however, the results were not transformed back into the original reference frame. There has been previous work that distinguishes between the dynamics inside and outside of focal adhesions, such as using FLIP [25] and FRAP [26] and Raster Image Correlation Spectroscopy [27].

Other focal adhesion detection methods use thresholding (such as a high pass filter) to find adhesions in an image, tracking their individual movement in time while collecting information on their size, shape and lifetime [21, 22]. However, these methods are unable to identify the internal dynamics, as well as they have difficulty tracking focal adhesions from frame to frame when neighboring adhesions are present and adhesion mergers or separations occur.

To illustrate the technique, we will analyze a migrating osteosarcoma cell expressing paxillin, a structural adaptor protein that interacts with integrins and focal adhesion kinase in focal adhesions [28]. Using two families of wavelets, we will detect focal adhesions and their internal dynamics as well as characterize the cell morphology and track the cell fronts of the protruding regions. Finally, we will apply WIMS to better understand the effect of actomyosin stability on the dynamic behavior of focal adhesions by contrasting the behavior of nonmuscle myosin IIB with a mutant counterpart in a Chinese hamster ovary cell. In the mutant case, we will quantitatively describe the changes in the internal dynamics of the actomyosin filaments that result in the breakdown of the front-back polarization of the cell. We find that we can decompose a heterogeneous image series to obtain dynamic information and extract specific features across multiple spatial scales spanning from the optical resolution limit to the cell morphology scale.

## Mathematical overview

### Wavelet transform of images

One convenient way to filter an image and select features at specific length scales is to use continuous wavelet transforms. For a two-dimensional microscopy image, the wavelet transform is obtained by integrating the image intensity function, *I*(**r**), in the following manner,

$$\begin{array}{c} T\left(a,b\right)=\frac{1}{a}\int_{-\infty }^{\infty }dr\;I\left(r\right)\psi^{*}(\frac{r-b}{a}), \end{array}$$ (1)

where *a* is a length scale parameter, **r** is the position vector in the original image, **b** is a position coordinate specifying the center of the wavelet function *ψ*(**r**), and the superscript * denotes a complex conjugate. The wavelet transform *T*(*a*, **b**) acts as a filter that selects features of the original image that vary on a length scale proportional to *a* around the point (**r** = **b**) (the function *ψ*(**r**) is localized near the origin). Numerous functions can be used as wavelets as long as they satisfy the following two conditions:

$$\begin{array}{c} \int_{-\infty }^{\infty }dr\;\psi \left(r\right)=0, \end{array}$$ (2)

which ensures that the wavelet transform of a constant (uniform) image vanishes and

$$\begin{array}{c} \int_{-\infty }^{\infty }dr\;\psi \left(r\right)\psi {\left(r\right)}^{*}<\infty , \end{array}$$ (3)

which guarantees that the wavelet is localized in space.

Wavelets are more than simple filters. The original image can be recovered by inverting the wavelet transform as follows,

$$\begin{array}{c} I\left(r\right)=\frac{1}{c_{\psi }}\int_{-\infty }^{\infty }db\int_{0}^{\infty }da\frac{1}{a^{4}}\;T\left(a,b\right)\psi (\frac{r-b}{a}), \end{array}$$ (4)

where *c*_*ψ*_ is an overall constant that depends on the form of the wavelet function [29]. This constant is defined as:

$$\begin{array}{c} c_{\psi }=\int_{-\infty }^{\infty }dr\;g\left(r\right)g{\left(r\right)}^{*} \end{array}$$ (5)

where

$$\begin{array}{c} g\left(r\right)=\int_{-\infty }^{\infty }dr\frac{\psi \left(r^{\prime }\right)}{\left|r-r^{\prime }\right|}. \end{array}$$

The inverse wavelet transform is only well defined if *c*_*ψ*_ is finite. This imposes one last wavelet condition,

$$\begin{array}{c} 0<c_{\psi }<\infty , \end{array}$$ (6)

called the admissibility condition. Discrete wavelet transforms [30], which uses a discretized wavelet function, can also be used in the same context.

Throughout this paper, we will use two types of continuous wavelet transforms based on two families of wavelet functions. The first one is a common, circularly symmetric and real wavelet function,

$$\begin{array}{c} \psi \left(\frac{r-b}{a}\right)=\left(2-{\left|\frac{r-b}{a}\right|}^{2}\right){\text{e}}^{-{\left|\frac{r-b}{a}\right|}^{2}/2} \end{array}$$ (7)

called the “Mexican hat” wavelet, also known as the “Ricker” wavelet.

The second is a family of “stretched” wavelets that are extended in one direction:

$$\begin{array}{lll} \psi_{\xi ,\theta }\left(\frac{r-b}{a}\right) & = & \left(2-{\left(\frac{r_{∥}\left(\theta \right)-b}{a}\right)}^{2}-\frac{1}{\xi^{2}}{\left(\frac{r_{⊥}\left(\theta \right)-b}{a}\right)}^{2}\right)\times \\ & & {\text{e}}^{-\frac{1}{2}\left({\left(\frac{r_{∥}\left(\theta \right)-b}{a}\right)}^{2}+\frac{1}{\xi^{2}}{\left(\frac{r_{⊥}\left(\theta \right)-b}{a}\right)}^{2}\right)}, \end{array}$$ (8)

where the elongation/compression of the wavelet along *r*_⊥_ is determined by *ξ* (*ξ* > 1 for elongation and 0 < *ξ* < 1 for compression) and where the angle *θ* specifies the orientation of the rotated wavelet about its center,

$$\begin{array}{ccc} r_{∥}\left(\theta \right) & = & x\cos\theta -y\sin\theta \\ r_{⊥}\left(\theta \right) & = & x\sin\theta +y\cos\theta , \end{array}$$ (9)

where *x* and *y* are the coordinates in the frame of the original image. All orientations can be described using *θ*’s ranging from 0° to 180° due to the stretched wavelet’s two-fold rotational symmetry. Wavelet transform coefficients obtained with the stretched wavelet, *T*_*ξ*,*θ*_(*a*, **b**), are obtained from Eq 1 with *ψ* replaced by *ψ*_*ξ*,*θ*_. Similarly, the original image can be recovered from Eq 4 with *ψ* and *T* replaced by *ψ*_*ξ*,*θ*_ and *T*_*ξ*,*θ*_ with *c*_*ψ*,*ξ*,*θ*_ = *π* (1 + *ξ*^2^)/2*ξ*. It is straightforward to show that both types of wavelets satisfy the conditions given by Eqs 2 and 3.

We decided upon these two wavelets because one is circularly symmetric (the Mexican hat wavelet) and the other is non circularly symmetric (the stretched wavelet). Stretched wavelets are derived from Mexican hat wavelets but with added anisotropy. They were used instead of the more common Morlet wavelet (a Mexican hat with a plane wave through it) since the Morlet wavelet can have multiple waves within if scales are not carefully set resulting in a comb-like effect with multiple peaks inside the wavelet, as opposed to the stretched wavelet, which only has a single peak in the center.

The use of ridgelets, a combination of Radon transforms and wavelets, is another way to obtain orientation information, particularly for edge detection applications. However, ridgelets, in contrast to wavelets, do not approach zero in all directions as *r* → ∞. We decided to use a wavelet since the goal was to detect adhesions of finite length, which is better suited to a wavelet basis in detecting and localizing them.

## Methods

### Wavelet transform and inverse transform

In practice, Eqs 1 and 4 are rewritten with a discrete mesh:

$$\begin{array}{c} T\left(a,b\right)=\frac{1}{a}\sum_{j,k}\Delta r\;I\left(r_{jk}\right)\;\psi^{*}(\frac{r_{jk}-b}{a}) \end{array}$$ (10)

and

$$\begin{array}{c} I\left(r\right)=\frac{1}{c_{\psi }}\sum_{j,k}\sum_{n_{\text{min}}}^{n_{\text{max}}}\Delta b\Delta a\frac{1}{a_{n}^{4}}\;T\left(a_{n},b_{jk}\right)\psi (\frac{r-b_{jk}}{a_{n}}), \end{array}$$ (11)

where **r**_*jk*_ and **b**_*jk*_ are the position coordinates of the *j*, *k* indices in the two-dimensional image and the wavelet function, respectively, and *n* is the scale index parameter where *n*_min_ and *n*_max_ are its minimum and maximum values. This reconstructs the image using a partial integration over *a* that includes only the scales of interest that lie between *a*_*n*_*min*__ and *a*_*n*_*max*__. The wavelet transform and inverse wavelet transform coefficients are oversampled in space due to the translation invariant/shift invariant nature of wavelets [31].

The pixel size and full image dimension of the original image are insufficient to calculate the wavelet transform coefficients at very small or very large length scales (i.e. the *a* in Eq 1) when using a discrete mesh. Wavelet transforms at small length scales were performed on a finer mesh for better numerical accuracy where 16 (4×4) mesh points all retain the value of the each original pixel (no interpolation). This is necessary since there are not enough elements in the original discrete representation to properly sample a continuous wavelet at small length scales. For improved computational efficiency in calculating the large length scale wavelet transforms, each image in the series was shrunk by a factor of four, reassigning the mean values of four (2×2) pixels to the new (coarser) mesh and this scaled down image was padded with zeros with a border of size 3*a*.

The calculation time of the wavelet transform *T*(*a*, **b**), was shortened by avoiding the integration of the whole image in Eq 1. The integrals were bounded by a box centred at **b** where the corners are at (*b*_*x*_ ± 3*a*, *b*_*y*_ ± 3*a*) since *ψ*(*a*, **b**) is nearly zero outside this box. An analogous approach was used in finding the inverse wavelet transform.

To reduce the calculation time, we implemented a parallel approach using graphical processing units (Dual Intel Sandy Bridge EP E5-2670, 8-core, 2.6 GHz, 20MB Cache, 115W). For the U2OS cell, the image series was of size 590 × 638 pixels × 150 frames, which involved the introduction of a finer mesh for small length scales, a full range of equidistant *a*’s, and/or a span of different angles/orientations for the stretched wavelet transform. We decomposed this data set into subsets of multi-dimensional arrays. The size of a data subset is dependent on the specific application, depending on whether we were calculating wavelet transforms or inverse wavelet transforms, small or large scales and if we were using a range of angles. The total number of elements from the array to be analyzed and the size of the resulting output could not exceed the allocated memory available on a processor. The most extensive computation we did was run on 50 processors for five days and most runs involved less than 20% of the maximum computational resources. Our code is available on GitHub (https://github.com/toplak/wims).

**Fig 1:**
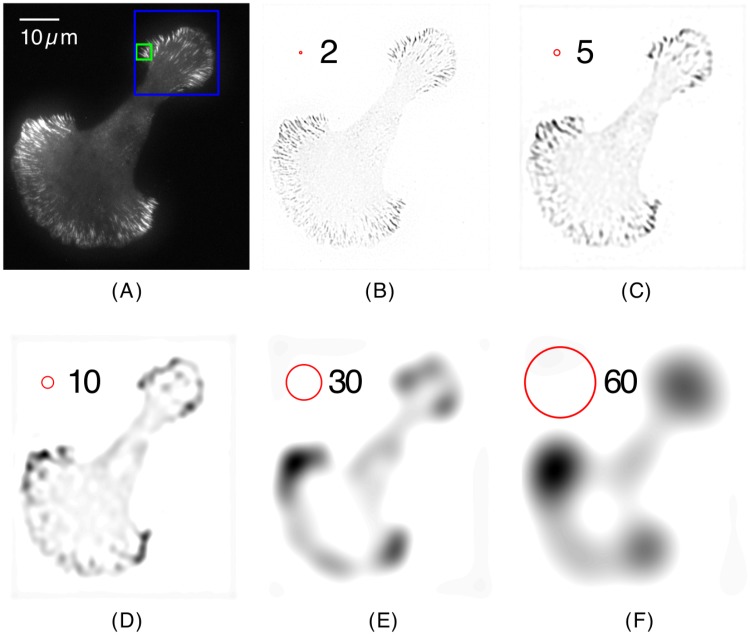
The continuous wavelet transform filters out different spatial scales. (*A*) Original TIRF microscopy image of paxillin-mKO in a U2OS cell plated on laminin and imaged at 37°C. (590×638 pixels, 0.105 *μ*m/pixel). The blue and green squares are regions of interest that are further analyzed in Figs 2 and 4. (*B–F*) Wavelet transform coefficients *T*(*a*, **b**) for multiple spatial scales, *a*, in ascending order: (*B*) *a* = 2 pixels, (*C*) *a* = 5, (*D*) *a* = 10, (*E*) *a* = 30, and (*F*) *a* = 60. The radius of the red circle in the upper left corner of each wavelet transform represents the value of *a*. The wavelet transform coefficients are presented in an inverted greyscale (regions of low/high intensity are lighter/darker).

**Fig 2:**
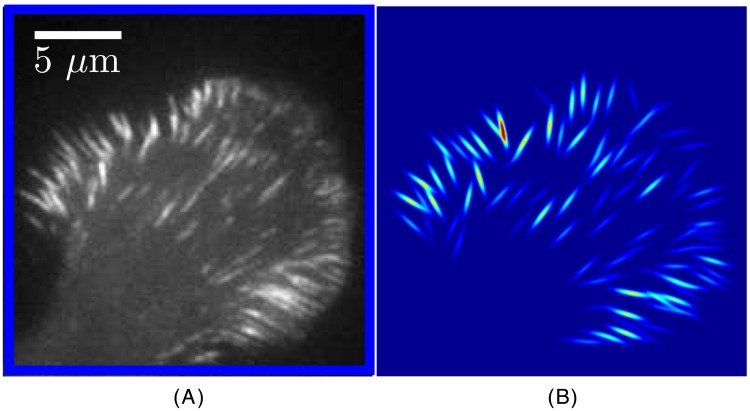
Wavelet mapping the location and orientation of the most prominent adhesions. (*A*) The region of the original image bound by the blue box in Fig 1*A*. (*B*) A spatial map of the amplitudes greater than 10% of the highest amplitude obtained by the wavelet transform across multiple orientations of stretched wavelets with parameters *a* = 10 and *ξ* = 1.5.

The Mexican hat wavelet transforms (such as those shown in Fig 1*B*–1*F*) were calculated for a range of scale parameters using Eq 10. Similarly, the stretched wavelet transforms were calculated using Eq 10 for a range of angles in one of two ways: (a) with a constant *a* and *ξ*, and (b) with variable *a* and *ξ*. For (a), the choice of *a* and *ξ* was determined by finding the set of adhesions with the highest wavelet coefficients for a range of values, but since the adhesions in the image are of variable lengths/widths, we selected an (*a*, *ξ*) pair that is the median value of the adhesions detected. For (b), we calculated the stretched wavelets for multiple (*a*, *ξ*) pairs across a broad range of values. This is used to reconstruct the image (such as in Fig 2*B*) by calculating the local maxima of *T*_*ξ*,*θ*_(*a*, **b**) from each set of wavelet transform coefficients (in terms of **b** and *θ*) and performing the following sum over these local maxima,

$$\begin{array}{c} I_{R}\left(r\right)=\sum_{\ell }^{L}\Delta a\Delta b\frac{1}{a^{4}}\;T_{\xi ,\theta_{\ell }}\left(a,b_{\ell }\right)\psi_{\xi ,\theta_{\ell }}(\frac{r-b_{\ell }}{a}), \end{array}$$ (12)

where **b**_ℓ_ and *θ*_ℓ_ indicate the location of the ℓ local maxima, with the total number of local maxima indicated by *L*. In order to recreate the configuration of the adhesions in the original images, we devised an automated routine to map out the location, orientation and size of adhesions. The algorithm calculates the wavelet coefficients for multiple scales and angles and finds their local maxima. The local maxima are arranged in descending order with the adhesion with the largest maxima placed first. Additional adhesions are placed one at a time, ensuring that potential adhesions do not overlap with any previously established adhesions, and iterate through the list of all local maxima. Potential adhesions with large wavelet coefficients are given priority but will not be placed if a previous adhesion is established in the same region. Each adhesion’s region is defined as an oriented ellipse with major and minor axes of size *a* and *ξ*. Fig 2*B* shows the reconstructed image, *I*_*R*_(**r**), obtained from this procedure when the sum is performed over all maxima found that are at least 10% as high as the wavelet coefficient of the highest local maximum. The entire image of the cell is reconstructed in S1 Fig. The inverse wavelet transforms from both families used Eq 11. The boundary was determined by first calculating the wavelet transforms, followed by the inverse wavelet transform for the coarsest scales. We then applied a threshold to find the boundary. The choice of the smallest scale in the inverse wavelet transform will determine the degree of accuracy of the boundary when compared to the actual cell edge (the difference being less than or equal to *a*).

This process was carried out across all the frames in the image series in order to track the appearance and disappearance of each adhesion as well as any changes in position and orientation. We assumed that an adhesion found in consecutive frames was the same adhesion if the position of the adhesion in the second frame was within an elliptical region with major and minor axes of size *a* and *ξ*, centered at the first adhesion and their orientations were within 10° of each other as long as it was not already linked to a previous adhesion that had a larger wavelet coefficient.

The internal dynamics within the adhesions first used the results from the reconstruction of *T*_*ξ*,*θ*_(*a*, **b**) from a single frame. A one-dimensional profile of each adhesion was found by taking a cross-section centered at the position, **b**, of the local maxima along its orientation, *θ*, extending to the cell boundary on one side and an equal length in the opposite direction. This line, *x*(*t*), remained fixed in position and orientation (*t* = 0 in S2 and S3 Figs) as cross-sectional data from the original image series was collected for the 20 frames preceding and following this frame to investigate the dynamics. The data above the mean of the maximum and minimum intensity were fit to a function, *ϕ*(*x*, *t*), consisting of a mixture of two Gaussians:

$$\begin{array}{c} \phi \left(x,t\right)=A_{1}^{\prime }\left(t\right)e^{-{\left(x\left(t\right)-\beta_{1}\left(t\right)\right)}^{2}/2\alpha_{1}^{2}\left(t\right)}+A_{2}^{\prime }\left(t\right)e^{-{\left(x\left(t\right)-\beta_{2}\left(t\right)\right)}^{2}/2\alpha_{2}^{2}\left(t\right)} \end{array}$$ (13)

where the amplitude of the peaks are $A_{1,2}=A_{0}+A_{1,2}^{\prime }$, *A*_0_ is the minimum intensity, *α*_1,2_(*t*) is the standard deviation of each peak and *β*_1,2_(*t*) is the location of the center of each peak. We used a constrained minimization to fit Eq 13 to ensure the amplitudes of both peaks were greater than zero. Occasionally there are errors in the tracking algorithm when it flips between fitting one peak or two within an adhesion. When fitting velocities these stray peaks are ignored.

Three different methods were used in determining the size and number of peaks for the one-dimensional profiles of the myosin IIB/vinculin data. The first method smoothed the data and found the local extrema. The peaks were arranged in order of their prominence, that is, the largest difference in local maximum and both adjacent local minima. Differences were also found between local maxima located between an adjacent local maximum and the second nearest local maximum in case a peak has a double hump profile. These peaks were fit with a Gaussian or a mixture of two Gaussians (Eq 13) for each interval between each pair of local minima. The second method involved taking the second derivative of the smoothed data and finding the local extrema. Mexican hat wavelets were fit to the derivative peaks and the size of the original peaks were found by integrating these wavelets twice. Finally, a wavelet composed of the sum of two Lorentzian functions was used:

$$\begin{array}{c} \ell \left(x\right)=\frac{1}{\pi \gamma }{\left[1+{\left(\frac{x-x_{0}}{\gamma }\right)}^{2}\right]}^{-1}-\frac{1}{2\pi \gamma }{\left[1+{\left(\frac{x-x_{0}}{2\gamma }\right)}^{2}\right]}^{-1} \end{array}$$ (14)

where *γ* is the Lorentz term and *x*_0_ is the location of the center of the peak. This function meets the criteria of a wavelet since it has compact support $\left(\int_{-\infty }^{\infty }\left|\ell (x)\right|dx<\infty \right)$ and finite energy $\left(\int_{-\infty }^{\infty }{\left|\ell (x)\right|}^{2}dx<\infty \right)$. This wavelet is similar in shape to the Mexican hat except it has very long tails to ±∞. This is in order to minimize the effects of the two negative regions on each side of the peak. The fits that are coincidental with all three methods were kept in our analysis.

## 1 Results

### Cell morphology detection and delineation

A fluorescence microscopy image time series of a cell expressing fluorescently-labeled proteins will have structures and processes that span across multiple length and time scales. Our ability to measure these properties depends on resolution, field of view, time sampling and the brightness of the fluorescent probes tagged to the macromolecules of interest. For example, Fig 1*A* shows a U2OS cell (an osteosarcoma cell line, [32]) expressing the fusion construct paxillin-mKO (a focal adhesion-associated adaptor protein, [28]), which has been imaged via total internal reflection microscopy (see Supplementary Materials). In this image, several characteristic length scales can be observed: the overall size/shape of the cell as well as the length/width of focal adhesions down to the spatial resolution limit set by diffraction and aberrations. It is advantageous to analyze the time behavior of the cell components at each of these length scales independently. This can be done using the wavelet transforms introduced in the Mathematical Overview.

Fig 1*B*–1*F* shows the wavelet transform *T*(*a*, **b**) of the image of the cell in Fig 1*A*. These were obtained using the Mexican hat wavelet function, Eq 7, to calculate the continuous wavelet transform given by Eq 1. The small *a* wavelet transform, Fig 1*B*, reveals the fine details of the image while the larger scale transform, Fig 1*F*, outlines the overall morphology of the cell.

Fig 2 shows a side by side view of the original image (Fig 2*A*) and a reconstructed image (Fig 2*B*) composed entirely of stretched wavelets that are positioned and oriented with respect to the highest registration in their surroundings. The reconstructed image in Fig 2*B* is made up of 180 angles, *θ*, in increments of one degree, with *a* = 10 and *ξ* = 1.5 pixels. Stretched wavelets with higher wavelet transform coefficients (shown in red/yellow) detect the adhesions with the highest intensities.

**Fig 3:**
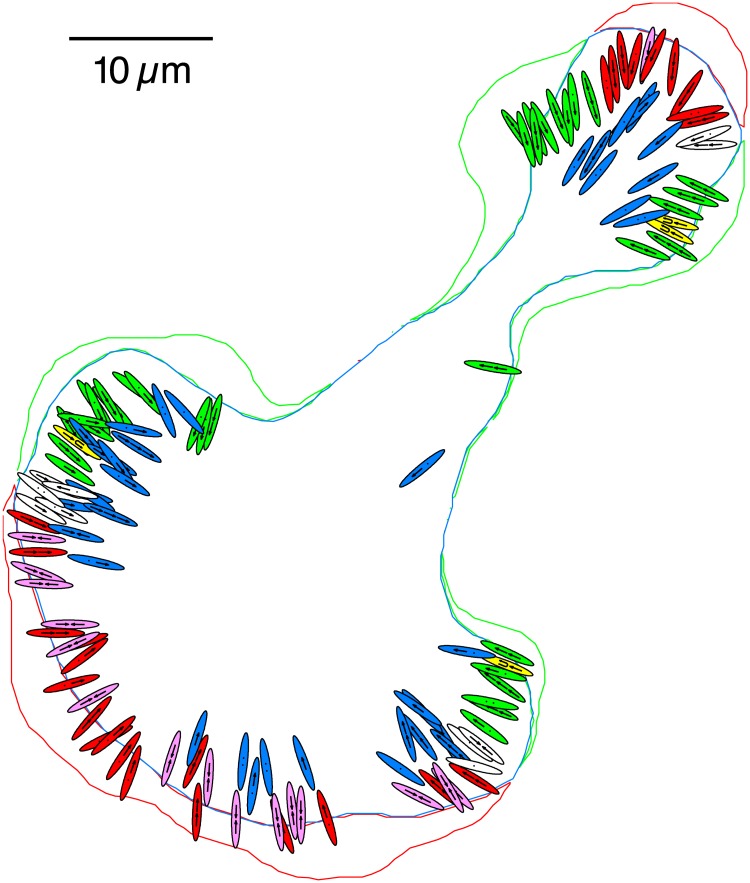
Multi-spatial scale cell information via wavelet analysis: Cell boundary tracking, detection of focal adhesions and internal dynamics. The movement of the cell boundary occurs over the course of 200 s (40 frames) where the green line is the boundary of the retracting region that the cell vacated, the red line is the boundary along the protruding region that the cell has entered and the blue line envelopes the region where the cell has remained over this time. The ellipses represent the focal adhesions detected at the midway point (100 s), and are enlarged by a factor of two to display the internal dynamics. The green and yellow ellipses are adhesions in the retracting region, red and pink adhesions are part of the protruding region, white adhesions are between the protruding and retracting adhesions yet still at the cell periphery and the blue are adhesions in the central region of the cell. The yellow and pink ellipses highlight special cases that are unique to each region. Each arrow and dot inside an ellipse represent the presence of a sub population within each adhesion, where arrows show the direction of motion of a translating sub population and dots show sub populations, which remain stationary.

We now show that the shape of the cell boundary can be obtained by inverting the wavelet transforms from a subset of the larger *a*’s, such as those shown in Fig 1*D*–1*F*. This is accomplished by using Eqs 10 and 11 where *a*_*n*_min__ = 20, *a*_*n*_max__ = 200 and Δ*a* = 4. The results of this procedure are depicted in Fig 3, which shows the outlines of the cell boundary for two images. In addition to the large scale features, Fig 3 also shows the configurations of the focal adhesions detected in the middle frame (color coded by region). In addition, the internal dynamics of each focal adhesion are visualized, where each symbol indicates a separate cluster of proteins inside a particular adhesion. The translational velocity of adhesions is small in comparison to the internal dynamics within the adhesions. The internal dynamics will be revisited later in this paper.

### Detection of focal adhesions

Focal adhesions are elongated and oriented along one direction (with their long axis roughly perpendicular to the cell boundary). In order to detect the focal adhesions we require a method to identify adhesions using scales that match their length, width and characteristic orientation. The stretched wavelet is used for this purpose (Eq 8).

**Fig 4:**
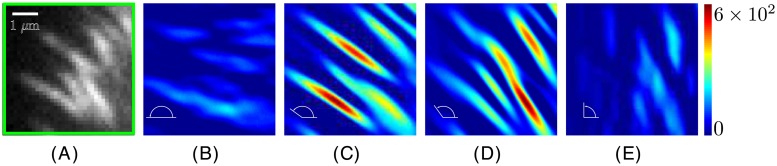
Stretched wavelet transform coefficients represented as heat maps for four different wavelet orientations. (*A*) 36×36 pixels image extracted from the green box in Fig 1*A*. (*B–E*) Stretched wavelet transform coefficients, having units of [pixels]^2^ of spatial frequency 1/(*aδr*). The parameters of the stretched wavelet were *a* = 10 and *ξ* = 1.5 with *θ* values of (*B*) *θ* = 180°, (*C*) *θ* = 144°, (*D*) *θ* = 126° and (*E*) *θ* = 90° (see Eq 8). Note how it maximally registers for those adhesions in the matched orientations, indicated by the red amplitude.

Fig 4 focuses on a small region of interest in the image (green box in Fig 1*A*) that shows seven focal adhesions that are similarly oriented. The wavelet transform of that region of the image is performed with the stretched wavelet with *a* = 10, *ξ* = 1.5 (the wavelet is over six times longer than its width) and at various orientations. Fig 4*C* and 4*D* show that the amplitude of the wavelet transform is large when the orientation of the focal adhesions and that of the wavelet function match. In contrast, the wavelet transforms shown in Fig 4*B* and 4*E* were performed with a stretched wavelet orientation that does not match that of any of the focal adhesions. Hence, the amplitude for these two panels is drastically reduced. Note that the procedure is capable of detecting slight variations in orientation of the focal adhesions. This is evident from the focal adhesion in the bottom right of Fig 4*A*, which has a slightly different orientation than the other focal adhesions in the rest of the image. This adhesion is brighter in Fig 4*D* than its neighbors, which appear brighter in Fig 4*C*. This means that the wavelet coefficients in this region are at their maximum at this slightly different orientation, (differing by 18° from Fig 4*C*). This adhesion is being picked up in both Fig 4*C* and 4*D*, but the coefficient is highest in Fig 4*C*. The wavelet coefficient at this position and angle has contributions from both the central adhesion and the neighboring adhesion on the same axis but it is still not as elevated as much as the same coefficient in Fig 4*C*. This additional contribution from the nearby adhesion would still be visible at other angles in the same range. The signal is maximized at a specific angle, but nevertheless detectable at others.

We can approximate and rapidly reconstruct the gross features of the focal adhesion distribution using the stretched wavelet coefficients across all angles (such as those shown in Fig 4) and assemble them as shown in Fig 2. The stretched wavelet transformed coefficients, *T*_*ξ*,*θ*_(*a*, **b**) are computed from a smaller region of interest in the cell image shown by the blue box in Fig 1*A* and here reproduced in Fig 2*A*. The family of stretched wavelets used to reconstruct the image in Fig 2*B* uses *a* = 6 pixels and *ξ* = 1 across 180 different angles, *θ*. The results show that by using the right wavelet (here, a stretched wavelet that is similar in shape to the focal adhesions), the main features of the original image on a given scale can be reconstructed with only a few wavelet coefficients. Moreover, the number of maxima that were found is *L* = 92, which is a measure of the number of focal adhesions in the image.

**Fig 5:**
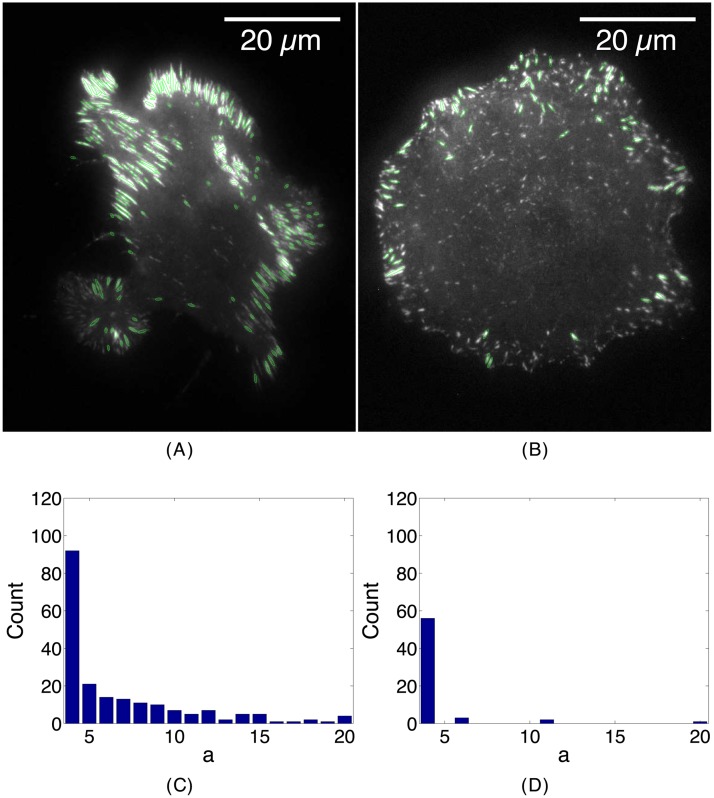
Finding adhesions of different sizes in U2OS cells using stretch wavelet transforms. (*A*) An image of a U2OS cell plated on the extracellular matrix protein laminin (10 *μ*g/mL). (*B*) An image of a U2OS cell plated on the extracellular matrix protein fibronectin (2 *μ*g/mL). Green ellipses show the detection of the position and the orientation of the adhesions found via stretch wavelets. The major and minor axes of each ellipse represent the length and width parameters, (*a*, *ξ*). (*C*) Histograms of the length scales (*a*) of the adhesions plated on laminin. (*D*) Histograms of the length scales (*a*) of the adhesions plated on fibronectin. Bin size 0.105 *μ*m (1 pixel) for both. Note that adhesions of longer lengths (*a*) as well as a greater number of total adhesions are detected in the cell plated on laminin compared to its fibronectin counterpart.

We can take this a step further by expanding the wavelet family to include a range of scale parameters *a* and *ξ* in calculating stretched wavelet transforms as shown in Fig 5. In Fig 5A and 5B we are able to reconstruct the gross features of the focal adhesion distribution of two U2OS cells: one plated on the extracellular matrix protein laminin (Fig 5A) and the other plated on the extracellular matrix protein fibronectin (Fig 5). In each image, the entire set of stretched wavelet transforms were calculated using all combinations of *a* = {4, 5, 6 … 19, 20} pixels and $\xi =\{\frac{1}{2},1,\frac{3}{2}\}$ pixels across 360 different angles, *θ*. In Fig 5A, *L* = 201 adhesions were found inside the cell plated on laminin, while in Fig 5B, *L* = 62 adhesions were detected on fibronectin. The adhesions in Fig 5A and 5B were binned by their scale, *a*, as shown in Fig 5C and 5D. Fig 5C shows that the majority of adhesions in Fig 5A were greater than the minimum scale (*a* = 4) and were detected across the entire range (*a* = 4 – 20), tapering off in number as the scale increased. This is in contrast to Fig 5D where nearly all of the adhesions (56/62) in Fig 5B were grouped in the minimum scale (*a* = 4).

We observe that the morphology of the fibronectin-plated cell (Fig 5B) is less polarized and active than its laminin-plated counterpart, which has an irregular boundary and contains larger adhesions (Fig 5A). The identification of different numbers and sizes in adhesions in U2OS cells on laminin and fibronectin by WIMS opens the possibility of establishing additional correlations with the level of cell activity and shape.

### Cell boundary and focal adhesion analysis

**Fig 6:**
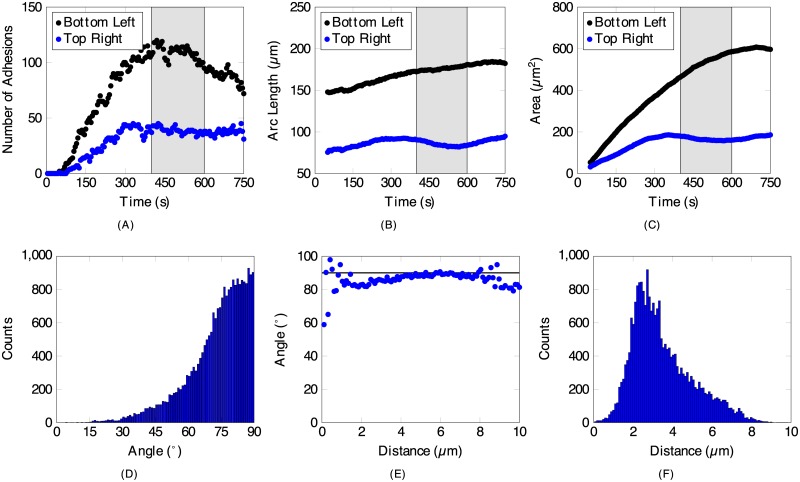
Dynamic information and distribution analysis from wavelet analysis. (*A*–*C*) highlights dynamic information obtained via wavelet analysis from (*A*) the adhesion scale, and (*B, C*) across the whole cell for the two protruding regions of the cell shown in Fig 3, with the region in the top right corner in blue and the region in the bottom right corner in black. The light gray background illustrates the time period pertaining to Fig 3, S2, S3 and S4 Figs. (*A*) The number of adhesions detected as a function of time. (*B*) The arc length of the cell fronts in the protruding region over time. (*C*) The area swept out by the protruding regions over time. (*D*–*F*) Information obtained from all the adhesions detected throughout the image series across all regions grouped into 100 bins. (*D*) The distribution of the angle between the orientation of an adhesion and the tangent line of the nearest cell boundary to the adhesion. The majority of adhesions are found to be perpendicular to its targeted cell boundary. (*E*) A plot of the mean angle in (*D*) separated by the distance between each adhesion and its corresponding cell boundary. The black line is at 90° (*F*) The distribution showing the prevalence of the adhesions categorized by distance in (*E*).

Fig 6*A* counts the number of adhesions detected, Fig 6*B* measures the arc length of the expanding cell front and Fig 6*C* measures the area of the two protruding regions shown in Fig 3, which are plotted as a function of time. The shaded regions of Fig 6*A*–6*C* shows the timeframe of the dynamics in Fig 3 relative to the entire image series. Initially (*t* = 1 – 300 s), both regions expand outwards rapidly before slowing down, with the bottom left front moving at 1.80±0.16 *μ*m/min and the top right front moving at 1.65±0.13 *μ*m/min, respectively, where the cell front velocities were found by tracking the movement of the cell boundary in the protruding region. The bottom left protruding region shown in black in Fig 6*A*–6*C* developed many adhesions during this time, peaking at 450 s, with the adhesions gradually decreasing for the remainder of the image series. The top right protruding region shown in blue stops protruding at the 300 s mark, with the number of adhesions, arc length and area all plateauing. In fact, the arc length and area both decrease slightly, due to the two sides retracting inward as shown in Fig 3. The number of adhesions during this downward trending interval remains constant, in contrast to the bottom left region that loses large and elongated adhesions. At the beginning of the image series (*t* = 1 – 300 s), we observe the number of adhesions, arc length and area to be linear (Fig 6*A*–6*C*). With this information, we find a relationship between the product of the velocity of the front, duration of activity and arc length with the number of adhesions each region has. This correlation results in a constant of 2.97 adhesions/*μ*m^2^ for the bottom left region and 0.70 adhesions/*μ*m^2^ for the top right region, with the larger bottom left region having a greater density of adhesions. Comparing between both regions, 4.3× as many adhesions are mobilized in the larger bottom left region compared to the top right region.

At a given time, we detected approximately 130 adhesions and we studied over 150 time frames in the entire image series, which we grouped into bins based on their orientation and distance to the cell boundary (Fig 6*D*–6*F*). Fig 6*D* is a histogram that shows the likelihood that an adhesion’s orientation will be normal to the tangent line of the cell boundary. The great majority are nearly perpendicular, where 54.0% are between 75–90° and 84.4% are between 60–90°. This is also noticeable in Fig 3 by the configurations of the adhesions across all regions relative to the tangent lines at the boundary. Fig 6*E* sorts the angles in Fig 6*D* into bins by the distance from the cell boundary to each adhesion before averaging each bin. Fig 6*F* shows the number of adhesions in each bin in Fig 6*E*. There are very few adhesions located close to the cell boundary (less than 1 *μ*m), but as the counts increase with the distance, the angle begins to approach 90°. Over half (51.7%) of the adhesions are located between 2.0–3.5 *μ*m away from the cell boundary, with the maximum number of adhesions (919) located at 2.8 *μ*m.

### Description of the internal dynamics of adhesive structures

Ten different types of internal dynamics were observed within the adhesions, as shown in Fig 3, with a few limited to specific regions of the cell. This is in contrast to the negligible translational motion of the adhesions as a complex. Each of the adhesions are color coded by region, with the colors matching the cell boundaries of the red protruding, green retracting and blue central regions. The white adhesions are uncategorized as they do not appear to distinctly belong to either the protruding or retracting regions. The pink adhesions highlight colliding adhesions in the protruding region and the yellow adhesions show examples of sub populations changing directions in the retracting regions. The protruding, retracting and central regions are equally well represented in Fig 3, with each of these regions containing between 39–42 adhesions. Refer to S1 Table for a complete breakdown of the number of occurrences of each. A selection of ten of the cases in S1 Table (in bold and italics) have been included in S2 Table to compare the velocities of the sub populations detected within the adhesions.

We can detect a wide range of behaviors inside the adhesions characterized by Fig 3, which we catalog in S2, S3 Figs, S1 and S2 Tables. We will highlight two cases in detail, one in the retracting region (Panel *D* in S2 Fig and Panel *D* in S3 Fig) and one in the protruding region (Panel *F* in S2 Fig and Panel *F* in S3 Fig). We plotted the trajectory and size of each cluster in S2 Fig where the center of each circle charts the cluster position at a given time (*β*_1,2_(*t*) in Eq 13), the radius represents the amplitude above the mean ($A_{1,2}^{\prime }(t)$) of the peak and the line thickness of each circle represents the width of the peak (*α*_1,2_(*t*)). S3 Fig shows the size of each sub population, using the amplitude and peak width information to find the area under each Gaussian peak (the intensity/size of the peak). The dotted lines show the position of the cell boundary in time. We define *i* as a unit of fluorescence intensity from the image. In the retracting region, Panel *D* in S2 Fig shows a case of one sub population (in green) moving at the same velocity as the cell boundary (0.29 *μ*m/min). Another cluster (in blue) initially moves towards the cell boundary (with a velocity of 1.08 *μ*m/min) before switching directions to match the velocity of the other cluster and the cell rear. The latter cluster stays relatively constant in size while the initially larger unidirectional cluster becomes smaller at a rate of 22 *i*/s. In the protruding region, Panel *F* in S2 Fig shows an example of two colliding clusters, where one cluster (in blue) translates away from the cell boundary (−0.42 *μ*m/min) while another cluster (in green) moves towards it (0.39 *μ*m/min). Before their collision at 30 s, they both increase in size (the blue cluster at 36 *i*/s and the green cluster at 24 *i*/s). However, immediately after the collision the green cluster loses over half its size before recovering, while the blue cluster continues its gains (possibly at the expense of the green cluster) before a sharp decrease in size.

The six cases presented in S2 and S3 Figs of the possible range of dynamics within adhesions shows we can quantify many aspects of their behavior. First of all, multiple sub populations can be separated within a single adhesion where we can then differentiate multiple forms of transport. Reliable quantitative estimates of the varying intensities in time helps gain insight into the growing or shrinking of sub populations (including their appearance/disappearance), which allows for direct comparisons between nearby members. It also registers the time when major events occur, such as collisions or changes in direction, as well as the duration a sub population is in a certain state. Finally, incorporating the cell boundary details allows for a useful comparison between the velocity of the cell front/rear and the internal velocities of the adhesions.

### Internal dynamics categorization

S4 Fig shows the regional distribution of velocities of the diverse sub populations inside the adhesions shown in Fig 3, where the internal velocities are separated into (*A*) the protruding region (26 red and 16 pink adhesions in Fig 3), (*B*) the retracting region (35 green and four yellow adhesions in Fig 3), (*C*) the central region (39 blue adhesions in Fig 3), and (*D*), the intersecting region adjacent to both the protruding and retracting regions (the ten white adhesions in Fig 3). The red bars on the positive side represent the number that move away from the cell boundary, while the blue bars represent those that move in the direction towards the cell boundary. These sub populations are sub-adhesion clusters identified by fitting a two Gaussian mixture, not the adhesion itself. The majority of the sub populations in all of the regions consistently move away from the cell boundary, with 62/82 (76%) in the protruding region and 71/83 (86%) in the retracting region. This is even the case in the central region (Panel *C* in S4 Fig) where the separation between an adhesion and cell boundary is at least 4 *μ*m (60/80 = 75%). There are many more directed towards the cell boundary in Panel *A* in S4 Fig, the protruding region, than there are in the other regions of the cell, due to the 16 colliding cases (the pink adhesions in Fig 3) with a mean velocity of −0.52 ± 0.12 *μ*m/min (refer to S2 Table). The four cases with switching behavior in Panel *B* in S4 Fig, the retracting region (the yellow adhesions in Fig 3), are present with a mean velocity of −0.57 ± 0.33 *μ*m/min (S2 Table). Overall, the positive velocities tend to be larger in the retracting region compared to the protruding and central regions, having twice as many translating at a velocity between 0.5 to 1.0 *μ*m/min. The protruding and central regions contain many more velocities near zero, although there appears to be a slight yet noticeable translation away from the cell boundary in the protruding region as evidenced by the higher ratio of red to blue sub populations around zero in Panel *A* in S4 Fig compared to the central region that by definition is distant from any cell boundary, evident from the nearly equal likelihood it would lean positive or negative for stationary cases. This result indicates that large adhesions can undergo a small translation away from the boundary, which may be due to the drag of retrograde actin flow. This could be tested by extending the approach to two color channels and measuring the actin flow simultaneously [33].

### WIMS quantitatively describes the alterations to the internal dynamics of actin and adhesions induced by the introduction of a point mutation in NMII-B

**Fig 7:**
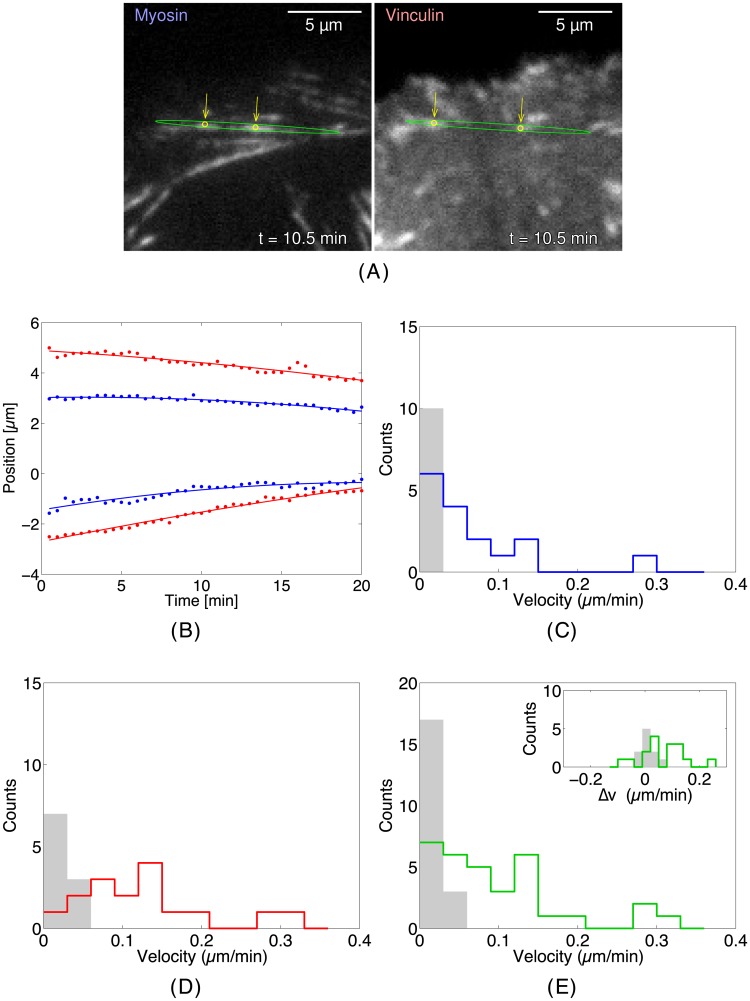
Internal dynamics of the actin filaments in a CHO.K1 cell expressing a mutant NMII-B. (*A*) The images of the myosin channel (left column) and the vinculin channel (right column) in the mutant myosin IIB cell at *t* = 10.0 min. The green ellipse shows the detection of the position and the orientation of the actin filament found via stretch wavelets and the yellow arrows pointing to yellow circles show the internal peaks of both the myosin and vinculin populations. (*B*) The trajectories of the myosin and vinculin poles pulling inwards for the example in (*A*). The vinculin poles are shown in red and the myosin poles are shown in blue. A line of best fit is included for each population. (*C*) Histograms of the myosin IIB velocities for the mutant (shown in blue) and the wild type case (shaded light gray). (*D*) Histograms of the vinculin velocities for the mutant (in red) and the wild type case (shaded light gray). (*E*) Histograms of the combined myosin IIB and vinculin velocities for the mutant (shown in purple) and wild type cases (shaded light gray) for all actin filaments. The velocities in the mutant case are faster than the wild type. The inset shows the histogram of the difference in velocities between a vinculin/myosin pair (${\vec{v}}_{v}-{\vec{v}}_{m}$) for both the mutant case (shown in green) and the wild type case (shaded light gray), where the vinculin velocities for the mutant cell are consistently higher than the myosin velocities. Bin size 0.03 *μ*m/min for both.

We now apply WIMS to a Chinese Hamster Ovary (CHO.K1) cell that expresses a mutant myosin II-B form (1935D) that displays decreased filament stability [34] compared to its wild type control to quantify the internal dynamics of the actin/myosin interaction and their effect over adhesion dynamics. In Fig 7*A* and 7*B* we show a typical actin filament in the cell expressing the 1935D mutant of NMII-B. Fig 7 illustrates how we extract information about the internal dynamics by first identifying an adhesion situated at a specific position and orientation using stretch wavelets. From this, we take cross-sections along the major axis of the adhesion in time for both vinculin and myosin (across both channels) to detect and characterize the dynamics of different populations for each protein. Fig 7*A* depicts, on the left, the distribution of the NMII-B 1935D mutant in a CHO.K1 cell devoid of endogenous NMII-B with superimposed ellipses to represent the spatially resolved adhesion found from the stretch wavelet transforms. The right panel represents the localization of vinculin in the same area. This panel also depicts a graphical representation of the determination of the center of mass of each separate protein population at a given time. In general, vinculin illuminates the adhesions that decorate the ends of the myosin II-defined actomyosin cables that support the adhesions. When examined in a time-lapse image series, we find that the centers of mass of vinculin along a straight line defined by the centers of mass of the myosin II move towards each other following a linear path, suggesting that they are part of the same larger-scale structure (Fig 7*B*). These measurements also allow us to estimate the velocity of these populations (Fig 7*B*).

Fig 7*C* and 7*D* show the velocity histograms obtained for adhesions in CHO.K1 cells expressing the wild type and 1935D NMII-B forms for myosin IIB (Fig 7*C*) and vinculin (Fig 7*D*). In Fig 7*E*, we combine the myosin and vinculin velocities for each cell type and partition them into a histogram. The average velocity of the mutant anchors is nearly five times the velocity of the wild type (0.10±0.07 *μ*m/min vs. 0.022±0.015 *μ*m/min). Wild type NMII-B is very slow, with an average velocity of 0.016±0.010 *μ*m/min, moving inwards. As is the case in the mutant cell, vinculin is faster than the myosin (0.028±0.017 *μ*m/min vs. 0.016 *μ*m/min).

The differences in velocity between vinculin and myosin populations (Δv = v_*v*_ − v_*m*_) that are found at the same pole are shown in the inset of Fig 7*E*. In the wild type case, Δv is clustered around zero, while in the mutant case, vinculin is faster than the 1935D mutant, resulting in most trajectories appearing like that shown in Fig 7*B* and the center of mass of vinculin gets closer to the center of mass of the 1935D by moving at twice the speed on average (0.13±0.07 *μ*m/min vs. 0.07±0.07 *μ*m/min).

Although it was previously known that the NMII-B 1935D mutant negatively affected the stability of actomyosin bundles from the overall lack of front-back polarization of the cell and that the NMII-B filaments turned over frequently [34], the mechanisms responsible were not specifically identified. With WIMS, we observe that both mutant and wild type cells have detectable populations of both vinculin and myosin at both ends of the actomyosin filaments. In the wild type case, these populations remain stationary, whereas in the mutant case, the filaments are no longer locked in place, resulting in all of the populations at the poles to move inward.

## Discussion

Wavelet imaging at multiple scales is a technique that combines several individual functions into a single tool by its ability to decouple cell dynamics and features across multiple spatial scales. At fine scales WIMS is a technique that can analyze diverse behavior of multiple clusters existing in the same focal adhesion to coarse scales where it can reveal large scale details such as the relationship between the number of adhesions and the cell boundaries. This approach provides insight into the mechanisms governing individual focal adhesions (both macroscopically and internally), as well as collectively based on type and region. Finally, we can integrate the information acquired across different scales to discover relationships such as that between the cell boundary velocity and the adhesion dynamics (Panels *A,B,D* in S2 Fig).

There is a noticeable difference in the internal dynamics of the actomyosin bundles between the wild type and mutant variant CHO.K1 cells, which may be explained by considering the stability of the filaments themselves. The coupling of the adhesive strength of the focal adhesions to the substratum and the interaction between actin and myosin that takes place at the filaments that define the adhesions generally results in a state of dynamic equilibrium, resulting in very slow inward movement of the adhesion and the filaments themselves. The data presented here show that reducing the stability of the filaments by introducing a mutation to myosin II promotes an imbalance that results in a strong inward movement of both the myosin II and the adhesion. The mutant myosin II-B mini filaments are less stable and consequently more easily pulled by the retrograde flow of the filament.

The subsequent shortening of the mutant NMII-B bundles is likely due to increased susceptibility to depolymerization of the actin filaments that underlie the myosin II-B bundles, which would be a consequence of the reduced stability of the overall structure. This is supported by evidence that indicates that unravelling of the actomyosin bundles render the filaments more susceptible to depolymerization [35].

Although the movement of the wild type NMII-B is almost negligible, some centripetal movement remains, indicating that, by default, actomyosin bundles are not in perfect steady-state, undergoing modest disassembly over time. The range of velocities for both the myosin and vinculin dynamics in the wild type cell are on the same order with the magnitudes found using STICS [36], where the actin and vinculin magnitudes were approximately 0.05 *μ*m/min.

In the U2OS cell from Fig 1, adhesions in the protruding and retracting regions share some similar dynamics, however key differences were (a) the presence of separate colliding populations in adhesions in protruding regions and (b) two populations initially colliding but then one population changes direction in retracting regions. Previous methods have not been able to distinguish between multiple populations within adhesions, and when using Fourier methods only the dynamics of the dominant peak is observed, or in the case of each population having similar size yet moving in opposite directions, they would negate each other [37]. Our results point to a more complicated system than simple sliding and treadmilling models provide.

Our technique is able to take an image series of a heterogenous cell and break it down into its base components visualized via fluorescence: the cell boundary, focal adhesions and the structures within the adhesions (Fig 3). It serves as a multi-purpose tool when previously a large set of separate techniques would be needed to cover a wide range of spatial and temporal resolutions. Techniques such as photon-counting histogram [27] and number and brightness analysis [38] measure dynamics, aggregation levels and the exchange of adhesion components, but do not bridge to the larger scale of the entire cell. A combination of approaches may be useful where WIMS targets the molecular measurements to key areas of dynamic turn over during cell migration.

In Fig 5, WIMS characterized the expected differences between adhesions on both a laminin and fibronectin substrate. We have previously described the contrasting behaviours of cells on different substrates [39]. In that study, we investigated the role of different integrin receptors and substrates in the control of the adhesive and migratory mechanisms for various cell types, including CHO.B2 and U2OS cells. We found that cells plated on laminin migrate and protrude more quickly than those plated on fibronectin. In particular, U2OS cells spread and migrate spontaneously on laminin with their adhesions elongating quickly, in comparison to these cells adhering to fibronectin, both due to integrins [39]. Fig 5 showed that WIMS can characterize adhesions regardless of their size or substrate and can quantify their contrasting biologically relevant behaviours.

Spatio-temporal image cross correlation analysis [40] cannot distinguish between the dynamics within adhesions and outside them unless they occur on different time scales. It compartmentalizes a data set into a grid, where each region of interest is analyzed using windowed Fourier transforms, which may lead to artifacts such as ringing. This is particularly problematic at the edges (regions with a sharp drop in intensity), especially at protruding edges, due to significant spatial heterogeneity. WIMS can be used on the same microscopic data series as the Fourier techniques. Since there are potentially unlimited families of wavelets, which have their own built-in parameters (e.g. orientation for stretch wavelets), as well as a range of scales, *a*, it is important to adapt your choice of parameters for your particular purpose or application. Narrowing down your possibilities will alleviate or reduce the longer computation times required for analysis.

Recent advances in super-resolution microscopy, such as photoactivation localization microscopy [41, 42], scanning angle interference microscopy [43] and more recently patterned-activation nonlinear structured illumination microscopy [44] have emerged allowing us to image individual molecules and sub-regions of adhesions across an entire cell. Since WIMS is a mathematical technique, it can be used together with super-resolution techniques.

Wavelets are an effective method to characterize the perimeter and area of a cell in tracking the movement of the boundary as well as any changes in cell shape. The results are comparable to those achieved using level set methods [45], a numerical method that involves first knowing the morphology of a cell, which is used together with a propagating front to iteratively fit to the contours of the cell over all of the frames [46]. Although the wavelet method worked well, the morphology of the U2OS cell was smooth and continuous, and would have inferior results compared to the level set method in cases of boundaries with steep discontinuities such as those found in neurons, as well as cell boundaries translating with wildly changing velocities. This is a shortcoming of the choice of wavelet, not WIMS; selecting or designing a wavelet that incorporates such discontinuous features (such as any from the Daubechies family [31] would have comparable results to those obtained from level set methods.

In this paper, we illustrated the power of wavelet transforms to analyze an image time series of a cell and obtained a wealth of information on the cell’s dynamics at different spatial scales. We have shown that wavelet transforms can be used to study the dynamics of cell morphologies, focal adhesion disassembly and formation as well as the microscopic dynamics that drive these larger scale processes. The translational motion of the focal adhesions can be tracked with wavelets as a comparison tool to the internal dynamics of the multiple clusters within the adhesions. We are able to separate individual adhesions that are clustered together and isolate their dynamics. We have the ability to classify adhesions based on their locations in the cell and their internal dynamics.

Here we have demonstrated some of the capabilities of the method as applied to a migrating cell in a simple tissue culture system, and to characterize dynamic differences for cells expressing a mutant form of myosin II-B relative to wild type. A great promise of this approach is that it can be readily extended beyond just spatial wavelets and generalized to two or more detection channels. These extensions will enable studies that can correlate information from multiple molecular constituents and bridge to the scale of the organelle and cellular level output behavior. We are currently working on such applications.

## Supporting information

S1 Fig Image reconstruction. Reconstructed image of the entire cell shown in Fig 1*A* using stretched wavelets of fixed size and shape (*a* = 10 and *ξ* = 1.5) and 180 orientations. The 250 largest wavelet transform coefficients were used. 590×638 pixels, 0.105 *μ*m/pixel, frame 80 of 150.
(EPS)

S2 Fig Plots representing the shape and position of the wavelet peaks detected within the adhesions as a function of time. The dashed line is the position in time of the cell boundary each adhesion is oriented towards. The colors blue, green and red are used to distinguish between different clusters, with each color matching its corresponding intensity in S3 Fig. The radius of the circle represents the peak amplitude and the width of the line of each circle is the peak width. The cases are organized into protruding (*A*–*D*) and retracting regions (*E* and *F*). (*A*) Two stationary peaks, where the velocity of both stationary clusters and the cell rear is the same at 0.04 *μ*m/min and the green peak closer to the cell boundary decreases in size while the blue peak increases in size. (*B*) Two translating peaks, moving away from the initial position of the cell boundary (the clusters and the boundary all moving at 0.45 *μ*m/min). (*C*) Three successive translating peaks, each moving away from the cell boundary at 0.47 *μ*m/min (green), 0.68 *μ*m/min (blue) and 0.63 *μ*m/min (red). (*D*) Two peaks that initially collide but soon after the blue peak changes direction, resulting in both moving away from the boundary. (*E*) One stationary peak and one translating peak (moving away at 0.08 *μ*m/min moving from the cell boundary, which moves in the opposite direction (at −0.27 *μ*m/min). (*F*) Colliding peaks. Refer to S3 Fig for corresponding intensity plots for each of these sub populations.
(EPS)

S3 Fig Plots showing the intensity contribution of the sub populations within each adhesion over time corresponding to the six cases presented in S2 Fig. The colors of each case match the shape and position of the clusters in S2 Fig. (*A*) Two stationary sub populations. The sub population nearest to the boundary (in green) decreases in size by 5 *i*/s, while the sub population furthest from the boundary (in blue) increases by 6.5 *i*/s, eventually overtaking the green cluster in size. This may be evidence of component exchange between sub populations, as there is no translation of either cluster although one cluster grows while the other one shrinks, with the net result of proteins being transported away from the cell boundary. (*B*) Two sub populations moving away from the cell boundary. The leading cluster (in green) remains the same size at the beginning, whereas the trailing cluster (in blue) initially grows, plateauing at double the size of the leading cluster, before both clusters drop at a rate of 40 *i*/s at approximately the same time. (*C*) Three sub populations moving away from the cell boundary. The three successive clusters each grow at a rate of approximately 40 *i*/s and then immediately diminish in size. (*D*) One sub population (in green) moving away from the cell boundary and one (in blue), which initially moves towards the cell boundary but changes course and finally moves away from it. (*E*) One stationary sub population (in green) and one (in blue) moving away from the cell boundary, which moves in the opposite direction (at −0.27 *μ*m/min). The translating cluster (in blue) grows at 20 *i*/s before diminishing in size at 18 *i*/s. The stationary cluster (in green) grows at 12 *i*/s before it also drops in size at 13 *i*/s. The blue cluster plateaus approximately 15 s before the green cluster. (*F*) Two colliding adhesions, one (in green) moving towards the cell boundary and one (in blue) moving away from the cell boundary. We define *i* as a unit of fluorescence intensity from the image.
(EPS)

S4 Fig Regional distribution of the internal velocities of the adhesions shown in Fig 3. (*A*) protruding region, (*B*) retracting region, (*C*) central region, and (*D*) the intersecting region between the protruding and retracting regions. Positive velocities (shown in red) move away from the cell boundary while negative velocities (shown in blue) move towards the cell boundary. Bin size of 0.1 *μ*m.
(EPS)

S1 Table Internal dynamics classification. Classification of the multiple types of internal dynamics identified within the 130 adhesions highlighted in Fig 3 by the total number of occurrences in the cell followed by their frequency based on the region they occupy. The ten unique internal dynamic categories are: (*A*) Two peaks colliding with one another, (*B*–*D*) one, two or three peaks translating away from the cell boundary, (*E*) a stationary peak and a translating peak translating away from the cell boundary, (*F*) a stationary peak and a translating peak translating towards the cell boundary, (*G*–*H*) one or two stationary peaks, (*I*) two peaks moving in opposite directions resulting in a collision but the peak translating towards the cell boundary switches direction and the two peaks both move away from the cell boundary, and (*J*) a stationary peak with two peaks translating away from the cell boundary. An arrow pointing to the right, →, represents a translating peak moving away from the cell boundary and an arrow pointing to the left, ←, represents a translating peak moving towards the cell boundary. A vertically centered dot, ⋅, represents a stationary peak and a hooked arrow pointing to the right, ↪, represents a translating peak that switches direction after colliding with another peak. Additional velocity information for ten of the most prominent and prevalent cases (highlighted in italics and bold) is provided in S2 Table. Colliding peaks (*A*) are mainly found in protruding regions while the similar case of peaks that initially collide but translate together are exclusively found in retracting regions. Cases of a translating peak with another peak (*C* and *E*) are commonly found throughout the cell, whereas situations with three translating peaks (*D*) are absent from protruding regions.
(PDF)

S2 TableVelocities grouped by their internal dynamics classification.The mean velocities of the peaks found within adhesions for various categories of internal dynamics for the ten bolded and italicized cases in S1 Table organized by region (protruding, retracting or central). For the cases of multiple peaks translating in the same direction, the velocities are first arranged in descending order, ${\vec{\text{v}}}_{1}>{\vec{\text{v}}}_{2}>{\vec{\text{v}}}_{3}$, where the mean velocity is calculated among each subcategory. In the adhesion undergoing switching behavior, ${\vec{\text{v}}}_{1}$ is the unwavering peak and ${\vec{\text{v}}}_{2A/2B}$ are the velocities of the peak before and after it flips direction. The confidence boundaries are the 95% confidence limit of the mean using standard deviation.(PDF)

S1 File Live-cell imaging. Details of the live-cell imaging.
(PDF)

## References

[1] Elson EL, Magde D. Fluorescence correlation spectroscopy. I. Conceptual basis and theory. Biopolymers. 1974;13(1):1–27. 10.1002/bip.1974.360130102 4818131

[2] Schwille P, Meyer-Almes FJ, Rigler R. Dual-color fluorescence cross-correlation spectroscopy for multicomponent diffusional analysis in solution. Biophys J. 1997;72(4):1878 10.1016/S0006-3495(97)78833-7 9083691 PMC1184381

[3] Petersen NO, Höddelius PL, Wiseman PW, Seger O, Magnusson KE. Quantitation of membrane receptor distributions by image correlation spectroscopy: concept and application. Biophys J. 1993;65(3):1135–1146. 10.1016/S0006-3495(93)81173-1 8241393 PMC1225831

[4] Wiseman PW, Petersen NO. Image correlation spectroscopy. II. Optimization for ultrasensitive detection of preexisting platelet-derived growth factor-beta receptor oligomers on intact cells. Biophys J. 1999;76(2):963 10.1016/S0006-3495(99)77260-7 9916027 PMC1300045

[5] Comeau JWD, Costantino S, Wiseman PW. A Guide to Accurate Fluorescence Microscopy Colocalization Measurements. Biophys J. 2006;91(12):4611–4622. 10.1529/biophysj.106.089441 17012312 PMC1779921

[6] Wiseman PW, Squier JA, Ellisman MH, Wilson KR. Two-photon image correlation spectroscopy and image cross-correlation spectroscopy. J Microsc. 2000;200(Pt 1):14–25. 10.1046/j.1365-2818.2000.00736.x 11012824

[7] Digman MA, Brown CM, Sengupta P, Wiseman PW, Horwitz AR, Gratton E. Measuring Fast Dynamics in Solutions and Cells with a Laser Scanning Microscope. Biophys J. 2005;89(2):1317–1327. 10.1529/biophysj.105.062836 15908582 PMC1366616

[8] Kolin DL, Ronis D, Wiseman PW. k-Space image correlation spectroscopy: a method for accurate transport measurements independent of fluorophore photophysics. Biophys J. 2006;91(8):3061–3075. 10.1529/biophysj.106.082768 16861272 PMC1578478

[9] Singh AP, Krieger JW, Buchholz J, Charbon E, Langowski J, Wohland T. The performance of 2D array detectors for light sheet based fluorescence correlation spectroscopy. Opt Express. 2013;21(7):8652 10.1364/OE.21.008652 23571955

[10] Hebert B, Costantino S, Wiseman PW. Spatiotemporal image correlation spectroscopy (STICS) theory, verification, and application to protein velocity mapping in living CHO cells. Biophys J. 2005;88(5):3601–3614. 10.1529/biophysj.104.054874 15722439 PMC1305507

[11] Mount N, Tate N, Sarker M, Thorne C. Evolutionary, multi-scale analysis of river bank line retreat using continuous wavelet transforms: Jamuna River, Bangladesh. Geomorphology. 2013;183:82–95. 10.1016/j.geomorph.2012.07.017

[12] Daubechies I. Ten Lectures on Wavelets. SIAM; 1992.

[13] Boutet de Monvel J, Le Calvez S, Ulfendahl M. Image Restoration for Confocal Microscopy: Improving the Limits of Deconvolution, with Application to the Visualization of the Mammalian Hearing Organ. Biophys J. 2001;80(5):2455–2470. 10.1016/S0006-3495(01)76214-5 11325744 PMC1301433

[14] Sarder P, Nehorai A. Deconvolution methods for 3-D fluorescence microscopy images. IEEE Signal Proc Mag. 2006;23(3):32–45. 10.1109/MSP.2006.1628876

[15] Parton R, Davis I. Lifting the fog: Image restoration by deconvolution In: Celis JE, editor. Cell Biology—A Laboratory Handbook. Burlington: Elsevier Academic Press; 2006 p. 187–200.

[16] Yang H, Komatsuzaki T, Kawakami M, Takahashi S, Sibley RJ. Change-Point Localization and Wavelet Spectral Analysis of Single-Molecule Time Series In: Yang H, editor. Single-Molecule Biophysics: Experiment and Theory, Volume 146 Hoboken, NJ, USA: John Wiley & Sons, Inc; 2011 p. 217–243.

[17] Izeddin I, Boulanger J, Racine V, Specht CG, Kechkar A, Nair D, et al Wavelet analysis for single molecule localization microscopy. Opt Express. 2012;20(3):2081–2095. 10.1364/OE.20.002081 22330449

[18] Kobrinsky E, Mager DE, Bentil SA, Murata Si, Abernethy DR, Soldatov NM. Identification of Plasma Membrane Macro- and Microdomains from Wavelet Analysis of FRET Microscopy. Biophys J. 2005;88(5):3625–3634. 10.1529/biophysj.104.054056 15722423 PMC1305509

[19] Bekkers E, Duits R, Berendschot T, ter Haar Romeny B. A Multi-Orientation Analysis Approach to Retinal Vessel Tracking. J Math Imaging Vis. 2014;49(3):583–610. 10.1007/s10851-013-0488-6

[20] Möhl C, Kirchgessner N, Schäfer C, Hoffmann B, Merkel R. Quantitative mapping of averaged focal adhesion dynamics in migrating cells by shape normalization. J Cell Sci. 2012;125(1):155–165. 10.1242/jcs.090746 22250204

[21] Berginski ME, Vitriol EA, Hahn KM, Gomez SM. High-Resolution Quantification of Focal Adhesion Spatiotemporal Dynamics in Living Cells. PLOS ONE. 2011;6(7):e22025 10.1371/journal.pone.0022025 21779367 PMC3136503

[22] Broussard JA, Diggins NL, Hummel S, Georgescu W, Quaranta V, Webb DJ. Automated Analysis of Cell-Matrix Adhesions in 2D and 3D Environments. Sci Rep. 2015;5:8124 10.1038/srep08124 25630460 PMC4309964

[23] Lam Hui K, Wang C, Grooman B, Wayt J, Upadhyaya A. Membrane Dynamics Correlate with Formation of Signaling Clusters during Cell Spreading. Biophys J. 2012;102(7):1524–1533. 10.1016/j.bpj.2012.02.015 22500752 PMC3318117

[24] Jaqaman K, Loerke D, Mettlen M, Kuwata H, Grinstein S, Schmid SL, et al Robust single particle tracking in live cell time-lapse sequences. Nature Methods. 2008;5(8):695 10.1038/nmeth.1237 18641657 PMC2747604

[25] Wolfenson H, Bershadsky A, Henis YI, Geiger B. Actomyosin-generated tension controls the molecular kinetics of focal adhesions. J Cell Sci. 2011;124(9):1425–1432. 10.1242/jcs.077388 21486952 PMC3078811

[26] Le Dévédec SE, Geverts B, de Bont H, Yan K, Verbeek FJ, Houtsmuller AB, et al The residence time of focal adhesion kinase (FAK) and paxillin at focal adhesions in renal epithelial cells is determined by adhesion size, strength and life cycle status. J Cell Sci. 2012;125(19):4498–4506. 10.1242/jcs.104273 22767508

[27] Digman MA, Brown CM, Horwitz AR, Mantulin WW, Gratton E. Paxillin Dynamics Measured during Adhesion Assembly and Disassembly by Correlation Spectroscopy. Biophys J. 2008;94(7):2819–2831. 10.1529/biophysj.107.104984 17993500 PMC2267137

[28] Turner CE. Molecules in focus—Paxillin. Int J Biochem Cell B. 1998;30(9):955–959. 10.1016/S1357-2725(98)00062-4 9785458

[29] Farge M, Lewalle J, Schneider K. Wavelet Transforms In: Tropea C, Yarin A, Foss JF, editors. Handbook of Experimental Fluid Mechanics. Springer; 2007 p. 1378–1395.

[30] Addison PS. The Illustrated Wavelet Transform Handbook Introductory Theory and Applications in Science, Engineering, Medicine and Finance. CRC Press; 2010.

[31] Daubechies I. The wavelet transform, time-frequency localization and signal analysis. IEEE Transactions on Information Theory. 1990;36(5):961–1005. 10.1109/18.57199

[32] Raile K, Höflich A, Kessler U, Yang Y, Pfuender M, Blum WF, et al Human osteosarcoma (U-2 OS) cells express both insulin-like growth factor-I (IGF-I) receptors and insulin-like growth factor-II/mannose-6-phosphate (IGF-II/M6P) receptors and synthesize IGF-II: Autocrine growth stimulation by IGF-II via the IGF-I receptor. J Cell Physio. 1994;159(3):531–541. 10.1002/jcp.1041590317 8188767

[33] Ashdown GW, Cope A, Wiseman PW, Owen DM. Molecular Flow Quantified beyond the Diffraction Limit by Spatiotemporal Image Correlation of Structured Illumination Microscopy Data. Biophys J. 2014;107(9):L21–L23. 10.1016/j.bpj.2014.09.018 25418107 PMC4223199

[34] Juanes-Garcia A, Chapman JR, Aguilar-Cuenca R, Delgado-Arevalo C, Hodges J, Whitmore LA, et al A regulatory motif in nonmuscle myosin II-B regulates its role in migratory front—back polarity. J Cell Biol. 2015;209(1):23–32. 10.1083/jcb.201407059 25869664 PMC4395487

[35] Haviv L, Gillo D, Backouche F, Bernheim-Groswasser A. A Cytoskeletal Demolition Worker: Myosin II Acts as an Actin Depolymerization Agent. Journal of Molecular Biology. 2008;375(2):325–330. 10.1016/j.jmb.2007.09.066 18021803

[36] Brown CM, Hebert B, Kolin DL, Zareno J, Whitmore L, Horwitz AR, et al Probing the integrin-actin linkage using high-resolution protein velocity mapping. J Cell Sci. 2006;119(24):5204–5214. 10.1242/jcs.03321 17158922

[37] Kolin DL, Wiseman PW. Advances in image correlation spectroscopy: measuring number densities, aggregation states, and dynamics of fluorescently labeled macromolecules in cells. Cell Biochem Biophys. 2007;49(3):141–164. 10.1007/s12013-007-9000-5 17952641

[38] Digman MA, Wiseman PW, Choi C, Horwitz AR, Gratton E. Stoichiometry of molecular complexes at adhesions in living cells. PNAS. 2009;106(7):2170–2175. 10.1073/pnas.0806036106 19168634 PMC2630200

[39] Chen L, Vicente-Manzanares M, Potvin-Trottier L, Wiseman PW, Horwitz AR. The Integrin-Ligand Interaction Regulates Adhesion and Migration through a Molecular Clutch. PLOS ONE. 2012;7(7):e40202 10.1371/journal.pone.0040202 22792239 PMC3391238

[40] Toplak T, Pandzic E, Chen L, Vicente-Manzanares M, Horwitz AR, Wiseman PW. STICCS Reveals Matrix-Dependent Adhesion Slipping and Gripping in Migrating Cells. Biophys J. 2012;103(8):1672–1682. 10.1016/j.bpj.2012.08.060 23083710 PMC3475391

[41] Betzig E, Patterson GH, Sougrat R, Lindwasser OW, Olenych S, Bonifacino JS, et al Imaging Intracellular Fluorescent Proteins at Nanometer Resolution. Science. 2006;313(5793):1642–1645. 10.1126/science.1127344 16902090

[42] Hess ST, Girirajan TPK, Mason MD. Ultra-High Resolution Imaging by Fluorescence Photoactivation Localization Microscopy. Biophys J. 2006;91(11):4258–4272. 10.1529/biophysj.106.091116 16980368 PMC1635685

[43] Paszek MJ, DuFort CC, Rubashkin MG, Davidson MW, Thorn KS, Liphardt JT, et al Scanning angle interference microscopy reveals cell dynamics at the nanoscale. Nature Methods. 2012;9(8):825–827. 10.1038/nmeth.2077 22751201 PMC3454456

[44] Li D, Shao L, Chen BC, Zhang X, Zhang M, Moses B, et al Extended-resolution structured illumination imaging of endocytic and cytoskeletal dynamics. Science. 2015;349(6251):aab3500–aab3500. 10.1126/science.aab3500 26315442 PMC4659358

[45] Machacek M, Danuser G. Morphodynamic Profiling of Protrusion Phenotypes. Biophys J. 2006;90(4):1439 10.1529/biophysj.105.070383 16326902 PMC1367294

[46] Sethian JA. Level Set Methods and Fast Marching Methods Evolving Interfaces in Computational Geometry, Fluid Mechanics, Computer Vision, and Materials Science. Cambridge University Press; 1999.

