# Supplementary material for: Wavelet Imaging on Multiple Scales (WIMS) reveals focal adhesion distributions, dynamics and coupling between actomyosin bundle stability
Source: PLoS One. 2017 Oct 19;12(10):e0186058. doi: 10.1371/journal.pone.0186058 (PMC5648137; doi:10.1371/journal.pone.0186058)
Supplement: S1 Table — Classification of the multiple types of internal dynamics identified within the 130 adhesions highlighted in Fig 3 by the total number of occurrences in the cell followed by their frequency based on the region they occupy. The ten unique internal dynamic categories are: (A) Two peaks colliding with one another, (B–D) one, two or three peaks translating away from the cell boundary, (E) a stationary peak and a translating peak translating away from the cell boundary, (F) a stationary peak and a translating peak translating towards the cell boundary, (G–H) one or two stationary peaks, (I) two peaks moving in opposite directions resulting in a collision but the peak translating towards the cell boundary switches direction and the two peaks both move away from the cell boundary, and (J) a stationary peak with two peaks translating away from the cell boundary. An arrow pointing to the right, →, represents a translating peak moving away from the cell boundary and an arrow pointing to the left, ←, represents a translating peak moving towards the cell boundary. A vertically centered dot, ⋅, represents a stationary peak and a hooked arrow pointing to the right, ↪, represents a translating peak that switches direction after colliding with another peak. Additional velocity information for ten of the most prominent and prevalent cases (highlighted in italics and bold) is provided in S2 Table. Colliding peaks (A) are mainly found in protruding regions while the similar case of peaks that initially collide but translate together are exclusively found in retracting regions. Cases of a translating peak with another peak (C and E) are commonly found throughout the cell, whereas situations with three translating peaks (D) are absent from protruding regions. (PDF) [file pone.0186058.s005.pdf]

| | Internal Dynamics | Total | Protruding | Retracting | Ventral | Intersecting |
|---|---|---|---|---|---|---|
| $A$ | Colliding ( $\rightarrow\leftarrow$ ) | 18 | ***16*** | 0 | 1 | 1 |
| $B$ | 1 $\vec{\mathbf{v}}$ ( $\rightarrow$ ) | 13 | 2 | 4 | 6 | 1 |
| $C$ | 2 $\vec{\mathbf{v}}$ ( $\rightarrow\rightarrow$ ) | 32 | ***14*** | ***13*** | ***4*** | 1 |
| $D$ | 3 $\vec{\mathbf{v}}$ ($\rightarrow\rightarrow\rightarrow$) | 12 | 0 | ***6*** | ***5*** | 1 |
| $E$ | 1 $\vec{\mathbf{v}}$, 1 $\vec{\mathbf{0}}$ ( $\cdot$ $\rightarrow$ ) | 29 | ***8*** | ***10*** | ***9*** | 2 |
| $F$ | 1 $-\vec{\mathbf{v}}$, 1 $\vec{\mathbf{0}}$ ( $\leftarrow$ $\cdot$ ) | 4 | 0 | 0 | 3 | 1 |
| $G$ | 1 $\vec{\mathbf{0}}$ ( $\cdot$ ) | 7 | 0 | 0 | 5 | 2 |
| $H$ | 2 $\vec{\mathbf{0}}$ ( $\cdot$ $\cdot$ ) | 10 | 2 | 2 | 5 | 1 |
| $I$ | 1 switch, 1 $\vec{\mathbf{v}}$ ( $\rightarrow\hookrightarrow$ ) | 4 | 0 | ***4*** | 0 | 0 |
| $J$ | 2 $\vec{\mathbf{v}}$, 1 $\vec{\mathbf{0}}$ ( $\cdot$ $\rightarrow\rightarrow$ ) | 1 | 0 | 0 | 1 | 0 |
