# Supplementary material for: Wavelet Imaging on Multiple Scales (WIMS) reveals focal adhesion distributions, dynamics and coupling between actomyosin bundle stability
Source: PLoS One. 2017 Oct 19;12(10):e0186058. doi: 10.1371/journal.pone.0186058 (PMC5648137; doi:10.1371/journal.pone.0186058)
Supplement: S2 Table — The mean velocities of the peaks found within adhesions for various categories of internal dynamics for the ten bolded and italicized cases in S1 Table organized by region (protruding, retracting or central). For the cases of multiple peaks translating in the same direction, the velocities are first arranged in descending order, v→1>v→2>v→3, where the mean velocity is calculated among each subcategory. In the adhesion undergoing switching behavior, v→1 is the unwavering peak and v→2A/2B are the velocities of the peak before and after it flips direction. The confidence boundaries are the 95% confidence limit of the mean using standard deviation. (PDF) [file pone.0186058.s006.pdf]

| | Internal Dynamics | Velocities ($\mu$m/min) | | |
|---|---|---|---|---|
| Protruding | Colliding ( $\rightarrow\leftarrow$ ) | $\vec{\mathbf{v}}_1 = 0.44 \pm 0.09$ | $\vec{\mathbf{v}}_2 = -0.52 \pm 0.12$ | |
| | 2 $\vec{\mathbf{v}}$ ( $\rightarrow\rightarrow$ ) | $\vec{\mathbf{v}}_1 = 0.37 \pm 0.14$ | $\vec{\mathbf{v}}_2 = 0.17 \pm 0.05$ | |
| | 1 $\vec{\mathbf{v}}$, 1 $\vec{\mathbf{0}}$ ( $\cdot$ $\rightarrow$ ) | $\vec{\mathbf{v}}_1 = 0.22 \pm 0.13$ | $\vec{\mathbf{v}}_2 = 0.020 \pm 0.01$ | |
| Retracting | 2 $\vec{\mathbf{v}}$ ( $\rightarrow\rightarrow$ ) | $\vec{\mathbf{v}}_1 = 0.45 \pm 0.12$ | $\vec{\mathbf{v}}_2 = 0.21 \pm 0.07$ | |
| | 3 $\vec{\mathbf{v}}$ ($\rightarrow\rightarrow\rightarrow$) | $\vec{\mathbf{v}}_1 = 0.75 \pm 0.11$ | $\vec{\mathbf{v}}_2 = 0.55 \pm 0.14$ | $\vec{\mathbf{v}}_3 = 0.37 \pm 0.14$ |
| | 1 $\vec{\mathbf{v}}$, 1 $\vec{\mathbf{0}}$ ( $\cdot$ $\rightarrow$ ) | $\vec{\mathbf{v}}_1 = 0.24 \pm 0.10$ | $\vec{\mathbf{v}}_2 = 0.005 \pm 0.005$ | |
| | 1 switch, 1 $\vec{\mathbf{v}}$ ( $\rightarrow\hookrightarrow$ ) | $\vec{\mathbf{v}}_1 = 0.35 \pm 0.17$ | $\vec{\mathbf{v}}_{2A} = -0.57 \pm 0.33$ | $\vec{\mathbf{v}}_{2B} = 0.32 \pm 0.14$ |
| Central | 2 $\vec{\mathbf{v}}$ ( $\rightarrow\rightarrow$ ) | $\vec{\mathbf{v}}_1 = 0.47 \pm 0.16$ | $\vec{\mathbf{v}}_2 = 0.15 \pm 0.12$ | |
| | 3 $\vec{\mathbf{v}}$ ($\rightarrow\rightarrow\rightarrow$) | $\vec{\mathbf{v}}_1 = 0.43 \pm 0.16$ | $\vec{\mathbf{v}}_2 = 0.26 \pm 0.08$ | $\vec{\mathbf{v}}_3 = 0.17 \pm 0.02$ |
| | 1 $\vec{\mathbf{v}}$, 1 $\vec{\mathbf{0}}$ ( $\cdot$ $\rightarrow$ ) | $\vec{\mathbf{v}}_1 = 0.37 \pm 0.16$ | $\vec{\mathbf{v}}_2 = 0.010 \pm 0.02$ | |
