# Supplementary material for: Wavelet Imaging on Multiple Scales (WIMS) reveals focal adhesion distributions, dynamics and coupling between actomyosin bundle stability
Source: PLoS One. 2017 Oct 19;12(10):e0186058. doi: 10.1371/journal.pone.0186058 (PMC5648137; doi:10.1371/journal.pone.0186058)
Supplement: S1 File — Details of the live-cell imaging. (PDF) [file pone.0186058.s007.pdf]

# Supporting Information

## Live-Cell Imaging

U2OS cells (ATCC HTB-96) were imaged using total internal reflection fluorescence (TIRF) microscopy in an Olympus IX70 inverted microscope with a 1.45 NA (oil) PlanApo ×60 TIRFM objective (Olympus America Inc., Center Valley, PA), fitted with a Ludl modular automation controller (Ludl Electronic Products, Hawthorne, NY) and controlled by Metamorph software (Molecular Devices, Sunnyvale, CA). Monomeric Kusabira-Orange protein (mKO) was excited with a 543 nm He-Ne laser line. A polychroic mirror (Z488/543rpc), a dual emission filter (Z488/543) and HQ620/60 emission filters were used to acquire the mKO image series (Chroma Technology, Bellows Falls, VT). The samples were imaged on a Retiga CCD camera (Retiga Exi; Qimaging, Surrey, BC, Canada). The U2OS image series were recorded with a pixel size of 0.105 $\mu$m, an exposure time of 50 ms and $\delta$t = 5 s, consisting of 150 total frames. The image series in Fig. 1 was first analysed by other methods in (38).

CHO.K1 cells (ATCC CCL-61) co-expressing GFP-MIIB (wild type or 1935D) and mCherry-Vinculin were also imaged on an automated inverted microscope (IX83; Olympus) fitted with a 60× Apochromat N 60× OTIRF

(NA 1.49) objective, a cellTIRF Mitico Unit (Olympus), and 491/561-nm laser lines. Dual GFP-mCherry samples were visualized with a dual emission filter (Z488/561). Images were captured using an electron-multiplying CCD camera (ImagEM X2; Hamamatsu Photonics) and Xcellence software (Olympus). These image series were recorded with a pixel size of 0.138 $\mu$m, an exposure time of 150 ms and 300 ms for the GFP and the mCherry, respectively, and $\delta t = 30$ s, consisting of 150 total frames.
